# Ischemic Colitis Is a Risk Factor for Clostridium difficile Infection

**DOI:** 10.7759/cureus.26076

**Published:** 2022-06-19

**Authors:** Shrouq Khazaaleh, Adalberto J Gonzalez, Mohammad Alomari, Vaibhav Wadhwa, Bhavan Shah, Bo Shen

**Affiliations:** 1 Internal Medicine, Cleveland Clinic Foundation, Cleveland, USA; 2 Gastroenterology and Hepatology, Cleveland Clinic Florida, Weston, USA; 3 Gastroenterology, Cleveland Clinic Florida, Weston, USA; 4 Neurology, Division of Epilepsy, Penn State Health Milton S. Hershey Medical Center, Hershey, USA; 5 Gastroenterology and Hepatology, Columbia University Irving Medical Center, New York City, USA

**Keywords:** hospitalization outcomes, diverticulitis colon, gut dysbiosis, ischemic colitis, clostridium difficile

## Abstract

Introduction: Clostridium difficile infection (CDI) is an anaerobic infection that can carry detrimental outcomes for patients and is a growing burden to the US healthcare system. Various theories have been proposed for the etiopathogenesis of CDI, including antibiotic use, dysbiosis, and acid suppression. The role of ischemia in CDI has not been explored. We hypothesize that tissue ischemia is a risk factor for the development of CDI. The study aimed to assess whether ischemia was a risk factor for CDI using ischemic colitis as a target population.

Methods: We performed a case-control study using the National Inpatient Sample (NIS) database in 2013. The study group included all patients with ischemic colitis (ICD 9 Code: 557.0, 557.1, 557.9) and the control group included all patients with diverticulitis (ICD 9 Code: 562.11, 562.13). Univariable and multivariable analyses were performed to assess the risk factors associated with CDI (ICD 9 Code: 008.45). The case and control groups were compared using the chi-square test for analysis. Continuous variables were compared using t-tests and categorical variables were compared using Rao-Scott chi-square tests. In addition, multivariable logistic regression analysis was performed to assess the association between disease group and CDI while adjusting for confounders. Univariable analysis was performed to assess differences between subjects with ischemic colitis and those with diverticulitis; continuous variables were compared using t-tests and categorical variables were compared using Rao-Scott chi-square tests. All analyses were done using SAS (version 9.4, The SAS Institute, Cary, NC).

Results: We analyzed more than 30 million hospitalizations in 2013, with 120,490 being Ischemic colitis-related admissions and 309,940 being diverticulitis-related admissions. The rate of CDI was more in the ischemic colitis group than in the diverticulitis group (odds ratio [OR] = 1.39; 95% confidence interval [CI] [1.03-1.88], p=0.03). After adjusting for all variables, multivariate analysis showed CDI was associated with ischemic colitis (OR = 2.06; 95% CI 1.59-2.65, p<0.001).

Conclusion: CDI was shown to be more prevalent in ischemic colitis than in diverticulitis control in this population-based study. As C. difficile is an anaerobe, we hypothesize that tissue hypoxia is a risk factor for its development. Further studies are needed to validate our findings.

## Introduction

Clostridium difficile infection (CDI) is a major source of healthcare burden, including cost, morbidity, and mortality. It is the most common pathogen isolated in healthcare-associated infections, accounting for 12.1% [1], and the most common nosocomial infectious diarrhea [2]. Some of the most well-known risk factors for CDI that have been studied include antibiotics, acid-suppressing agents, age over 65 years, comorbid conditions, antineoplastic chemotherapy, and increased length of hospital stay [3].

The presence of tissue ischemia has been linked to CDI remotely but has not been extensively studied [4]. We hypothesized that ischemic colitis is a risk factor for the development of CDI for several reasons related to its pathogenesis that leave the colon vulnerable. Clostridium difficile is an anaerobic bacteria with spore formation that can survive hypoxic and acidic conditions. Ischemic colitis is a condition in which both of these factors are present in addition to decreasing innate intestinal defenses and causing microbiological dysbiosis. We aimed to assess whether ischemia was a risk factor for CDI using ischemic colitis as a target population.

The preliminary results of this article were presented in a poster form at the American College of Gastroenterology 2017 Annual Scientific Meeting (October 13-18 in Orlando) and published in the American Journal of Gastroenterology (October 2017, Volume 112, p S103).

## Materials and methods

We performed a case-control study using the National Inpatient Sample (NIS) database in 2013. The National Inpatient Sample is the largest all-payer inpatient database in the United States, and its large sample size is ideal for analyzing rare conditions and associations. No IRB is required by our institution for this research work.

Case group

Our case group included all patients ≥18 years old with an ICD-9 code indicating a discharge diagnosis of ischemic colitis. This included acute ischemic colitis (ICD 9 Code 557.0), chronic ischemic colitis (557.1), and unspecified vascular insufficiency of the intestine (557.9).

Control group

Our control group included all patients ≥18 years old with an ICD-9 code indicating a discharge diagnosis of uncomplicated diverticulitis. This included diverticulitis of the colon without mention of hemorrhage (ICD 9 Code: 562.11) and diverticulitis of the colon with mention of hemorrhage (562.13). We excluded patients with complicated diverticulitis.

Data collection

Once the patients with ischemic colitis were identified, an excel sheet was used to gather information regarding the frequency of the diagnosis of CDI (ICD-9 code 008.45) and the risk factors associated with CDI.

Statistical analysis

Univariable and multivariable analyses were performed to assess the risk factors associated with CDI (008.45). The case and control groups were compared using the chi-square test for analysis. Continuous variables were compared using t-tests and categorical variables were compared using Rao-Scott chi-square tests. In addition, multivariable binomial logistic regression analysis was performed to assess the association between disease group and CDI while adjusting for confounders. Univariable analysis was performed to assess differences between subjects with ischemic colitis and those with diverticulitis; continuous variables were compared using t-tests and categorical variables were compared using Rao-Scott chi-square tests. All analyses were done using SAS (version 9.4, The SAS Institute, Cary, NC).

## Results

Overall analysis

We analyzed more than 30 million hospitalizations in 2013. 120,490 patients were admitted for Ischemic colitis, which comprised the case group. The control group included 309,940 patients admitted for diverticulitis in 2013. CDI was diagnosed in 65/120,490 patients with ischemic colitis, with an incidence of 0.054%. CDI had an incidence of 0.039% (120/309,940) in patients with diverticulitis. The rate of CDI was significantly higher in the ischemic colitis group compared to the diverticulitis group (odds ratio [OR] = 1.39; 95% confidence interval [CI] [1.03 - 1.88], p=0.03).

Baseline characteristics of hospital demographics and patients with ischemic colitis, diverticulitis, and C. difficile

Baseline characteristics of C. Difficile patients were studied (Table 1). We compared baseline characteristics of ischemic colitis and diverticulitis patients. Females were more likely to have ischemic colitis (64% vs 58%; p<0.001). There were no significant differences in race, household income, or insurance. Ischemic colitis patients were more likely to fall under the class 3 (36.3%) and class 4 (29.6%) Diagnosis Related Group (DRG) risk of mortality subclass. Diverticulitis patients were more likely to fall under the class 1 (59%) and class 2 (23.5%) DRG risk of mortality subclass (Table 2).

**Table 1:** Patient and hospital demographics and outcomes for patients in ischemic colitis versus diverticulitis. DRG: Diagnosis-related group CDI: Clostridium Difficile infection

		Ischemic colitis	Diverticulitis	P-Value
Indicator of sex	Male	35.9%	42.2%	
	Female	64.1%	57.8%	<0.001
	Total	100.0%	100.0%	
Race	White	80.3%	78.3%	
	Black	10.1%	9.1%	
	Hispanic	7.3%	10.4%	
	Other	2.2%	2.2%	
	Total	100.0%	100.0%	<0.001
Median household income national quartile for patient ZIP Code	$1 - $38,999	26.0%	25.9%	
	$39,000 - $47,999	26.8%	26.2%	
	$48,000 - $62,999	26.0%	25.0%	
	$63,000 or More	21.3%	23.0%	<0.001
Primary expected payer	Medicare	68.3%	50.9%	
	Medicaid	7.7%	7.1%	
	Private including HMO	21.6%	39.2%	
	Other	2.4%	2.8%	<0.001
All Patient Refined DRG: Risk of Mortality Subclass	0	0.0%	0.0%	
	1	14.6%	59.0%	
	2	19.5%	23.5%	
	3	36.3%	12.7%	
	4	29.6%	4.8%	<0.001
Bed size of hospital (STRATA)	1	12.8%	16.5%	
	2	26.6%	28.2%	
	3	60.6%	55.4%	<0.001
Region of hospital	1	19.5%	21.0%	
	2	23.6%	22.8%	
	3	37.2%	38.9%	
	4	19.7%	17.4%	<0.001
Location/teaching status of hospital (STRATA)	1	9.9%	14.2%	
	2	39.8%	43.5%	
	3	50.4%	42.3%	<0.001
CDI	Present	100.0%	100.0%	0.03
Sepsis	Present	100.0%	100.0%	<0.001
Bowel perforation	Present	100.0%	100.0%	<0.001
Bowel surgery	Present	100.0%	100.0%	<0.001
Died during hospitalization	Dead	100.0%	100.0%	<0.001

**Table 2:** Patient and hospital demographics and outcomes for patients in ischemic colitis and CDI versus diverticulitis and CDI. CDI: Clostridium difficile infection.

		Ischemic colitis and CDI		Diverticulitis and CDI		P-Value	
		N %		N %			
Age	Mean (SD)	71 (11)		73 (8)			
Indicator of sex	Male	53.8%	45.8%				
	Female	46.2%	54.2%				
	Total	100.0%	100.0%		0.297		
Race (uniform)	White	69.2%	66.7%				
	Black	7.7%	4.2%				
	Hispanic	23.1%	20.8%				
	Other	0.0%	8.3%				
	Asian or Pacific Islander	0.0%	0.0%				
	Native American	0.0%	0.0%				
	Total	100.0%	100.0%		0.258		
Median household income national quartile for patient ZIP Code	$1 - $38,999	25.0%	25.0%				
	$39,000 - $47,999	8.3%	29.2%				
	$48,000 - $62,999	33.3%	29.2%				
	$63,000 or More	33.3%	16.7%		0.004		
Primary expected payer (uniform)	Medicare	61.5%	70.8%				
	Medicaid	15.4%	0.0%				
	Private including HMO	15.4%	25.0%				
	Other	0.0%	0.0%				
	Self-Pay	7.7%	4.2%				
	No Charge	0.0%	0.0%		0.0005		
All Patient Refined DRG: Risk of Mortality Subclass	1	7.7%	29.2%				
	2	15.4%	8.3%				
	3	38.5%	50.0%				
	4	38.5%	12.5%		0.00001		
Bed size of hospital (STRATA)	1	7.7%	12.5%				
	2	30.8%	41.7%				
	3	61.5%	45.8%		0.119		
Region of hospital	1	30.8%	16.7%				
	2	15.4%	20.8%				
	3	23.1%	50.0%				
	4	30.8%	12.5%		0.0012		
Location/teaching status of hospital (STRATA)	1	0.0%	8.3%				
	2	46.2%	45.8%				
	3	53.8%	45.8%		0.05		
Sepsis	Present	30.8%	33.3%				
	Absent	69.2%	66.7%		0.722		
Bowelperforation	Present	0.0%	4.2%				
	Absent	100.0%	95.8%		0.095		
Bowel surgery	Present	15.4%	4.2%				
	Absent	84.6%	95.8%		0.0076		
Died during hospitalization	Dead	7.7%	0.0%				
	Alive	92.3%	100.0%		0.002		
LOS	Mean (SD)	22 (24)		8 (8)			
Number of chronic conditions	Mean (SD)	7 (3)		8 (4)			
Total Charges	Mean	241908		65350			

Patients with CDI were significantly more likely to be older than patients without CDI (mean: 50 vs 48 years old; p=0.003). CDI was associated with an increased length of stay (mean: 5.39 days vs 4.55 days; p<0.001). Patients with CDI had higher odds of having ischemic colitis, diverticulitis, or sepsis as a diagnosis (p<0.001). CDI patients were less likely to undergo bowel surgery (0.2% vs 23%; p<0.001); however, not more likely to have bowel perforation. Patients with CDI diagnosis were more likely to have coagulopathy (1.5% vs 0.5%; p<0.05), and electrolyte abnormalities (37% vs 73%; p<0.05) during their hospital stay. We found that some chronic diseases were also associated with CDI. These included chronic anemia, Congestive heart failure, and hypertension. Patients with CDI had greater odds of death than patients without CDI (p<0.001) (Table 3).

**Table 3:** Univariate analysis comparing ischemic colitis versus diverticulitis in CDI positive patients. CDI: Clostridium difficile infection.

		No CDI	Percentage	CDI Present	Percentage	P- Value
Sepsis	Total	53565	3.067%	60	0.935%	
	Ischemic colitis	28570	23.710%	20	30.769%	0.19
	diverticulitis	24995	8.062%	40	33.333%	0.000
Bowel surgery	Total	81380	23.227%	15	0.234%	0.003
	Ischemic colitis	30535	25.340%	10	15.385%	0.07
	diverticulitis	50845	16.401%	5	4.167%	0.000
Bowel perforation	Total	6555	17.939%	5	0.078%	0.17
	Ischemic colitis	4910	4.075%	0	0.000%	0.116
	diverticulitis	1645	0.531%	5	4.167%	0.000
History of ischemic colitis	Total	1835	10.019%	0	0.000%	0.000
	Ischemic colitis	1485	1.232%	0	0.000%	1.000
	diverticulitis	350	0.113%	0	0.000%	1.000
Died	Total	22360	3.325%	5	0.078%	0.719
	Ischemic colitis	18140	15.054%	5	7.692%	0.117
	diverticulitis	4220	1.361%	0	0.000%	0.418
Age	Total	48.65	(+- 27.61)	50.21	(+-25.257)	0.000
	Ischemic colitis	67.65	(+-16.84)	71	(+-10.68)	0.572
	diverticulitis	62.51	(+-15.76)	72.58	(+-8.229)	0.001
Length of stay	Total	4.55	(+-6.655)	5.39	(+-7.918)	0.000
	Ischemic colitis	9.02	(+-13.684)	21.77	(+-24.061)	0.000
	diverticulitis	5.36	(+-5.740)	8.33	(+-8.320)	0.000
AIDS	Total	380	0.574%	0	0.000%	0.000
	Ischemic colitis	150	0.124%	0	0.000%	0.922
	diverticulitis	230	0.074%	0	0.000%	0.915
Alcohol abuse	Total	8740	0.598%	15	0.234%	0.855
	Ischemic colitis	15	0.012%	15	23.077%	0.052
	diverticulitis	8725	2.814%	0	0.000%	0.053
Iron deficiency anemia	Total	72950	1.385%	60	0.935%	
	Ischemic colitis	26185	21.730%	25	38.462%	0.002
	diverticulitis	46765	15.085%	35	29.167%	0.000
Rheumatoid arthritis	Total	15795	1.914%	5	0.078%	
	Ischemic colitis	4870	4.041%	0	0.000%	0.068
	diverticulitis	10925	3.524%	5	4.167%	0.417
Chronic blood loss anemia	Total	6635	0.889%	5	0.078%	
	Ischemic colitis	3290	2.730%	0	0.000%	0.165
	diverticulitis	3345	1.079%	5	4.167%	0.01
Congestive heart failure	Total	38635	1.500%	30	0.468%	
	Ischemic colitis	16870	14.000%	10	15.385%	0.427
	diverticulitis	21765	7.021%	20	16.667%	0.0000
Chronic pulmonary diseases	Total	83660	1.467%	20	0.312%	
	Ischemic colitis	28090	23.311%	0	0.000%	0.000
	diverticulitis	55570	17.925%	20	16.667%	0.404
Coagulopathy	Total	24065	1.551%	35	0.546%	
	Ischemic colitis	13965	11.589%	15	23.077%	0.007
	diverticulitis	10100	3.258%	20	16.667%	0.000
Depression	Total	49075	1.474%	30	0.468%	
	Ischemic colitis	15245	12.651%	5	7.692%	0.153
	diverticulitis	33830	10.912%	25	20.833%	0.001
Diabetes-uncomplicated	Total	77565	1.367%	50	0.779%	
	Ischemic colitis	23995	19.913%	20	30.769%	0.025
	diverticulitis	53570	17.280%	30	25.000%	0.017
Diabetes-complicated	Total	13785	0.967%	5	0.078%	
	Ischemic colitis	6040	5.012%	0	0.000%	0.035
	diverticulitis	7745	2.498%	5	4.167%	0.183
Drug abuse	Total	8815	0.607%	10	0.156%	
	Ischemic colitis	3015	2.502%	5	7.692%	0.024
	diverticulitis	5800	1.871%	5	4.167%	0.076
Hypertension	Total	250725	1.685%	125	1.949%	
	Ischemic colitis	77640	64.432%	35	53.846%	0.049
	diverticulitis	173085	55.830%	90	75.000%	0.000
Hypothyroidism	Total	62870	1.790%	5	0.078%	
	Ischemic colitis	19725	16.369%	0	0.000%	0.000
	diverticulitis	43145	13.917%	5	4.167%	0.002
Liver diseases	Total	17235	1.839%	15	0.234%	
	Ischemic colitis	6895	5.722%	10	15.385%	0.004
	diverticulitis	10340	3.335%	5	4.167%	0.372
Lymphoma	Total	3265	1.357%	0	0.000%	
	Ischemic colitis	1020	0.846%	0	0.000%	0.575
	diverticulitis	2245	0.724%	0	0.000%	0.418
Fluid and electrolyte disorder	Total	151225	2.037%	135	2.104%	
	Ischemic colitis	62380	51.768%	45	69.231%	0.004
	diverticulitis	88845	28.658%	90	75.000%	0.0000
Metastatic cancer	Total	7840	1.192%	5	0.078%	
	Ischemic colitis	3495	2.900%	0	0.000%	0.148
	diverticulitis	4345	1.402%	5	4.167%	0.028
Other neurologic disorder	Total	26335	1.126%	30	0.468%	
	Ischemic colitis	10455	8.676%	5	7.692%	0.5
	diverticulitis	15880	5.122%	25	20.833%	0.000
Obesity	Total	58755	1.567%	10	0.156%	
	Ischemic colitis	13340	11.071%	5	7.692%	0.26
	diverticulitis	45415	14.649%	5	4.167%	0.001
Paralysis	Total	6005	0.779%	0	0.000%	
	Ischemic colitis	3345	2.776%	0	0.000%	0.16
	diverticulitis	2660	1.666%	0	12.500%	0.355
Peripheral vascular disorder	Total	67300	3.793%	50	0.779%	
	Ischemic colitis	50770	42.133%	45	69.231%	0.000
	diverticulitis	16530	5.332%	5	4.167%	0.378
Psychoses	Total	14135	0.975%	0	0.000%	
	Ischemic colitis	5020	4.166%	0	0.000%	0.063
	diverticulitis	9115	2.940%	0	0.000%	0.028
Pulmonary circulation disorder	Total	9710	1.449%	15	0.234%	
	Ischemic colitis	4545	3.772%	0	0.000%	0.082
	diverticulitis	5165	1.666%	15	12.500%	0.000
Renal failure	Total	49315	1.351%	45	0.701%	
	Ischemic colitis	22020	18.274%	20	30.769%	0.01
	diverticulitis	27295	8.804%	25	20.833%	0.000
Solid tumor without metastasis	Total	8355	1.363%	0	0.000%	
	Ischemic colitis	3070	2.548%	0	0.000%	0.187
	diverticulitis	5285	1.705%	0	0.000%	0.122
Peptic ulcer diseases with no bleeding	Total	195	2.216%	0	0.000%	
	Ischemic colitis	95	0.079%	0	0.000%	0.95
	diverticulitis	100	0.032%	0	0.000%	0.962
Valvular diseases	Total	18445	1.689%	15	0.234%	
	Ischemic colitis	7205	5.979%	5	7.692%	0.349
	diverticulitis	11240	3.626%	10	8.333%	0.012
Weight loss	Total	36250	2.438%	50	0.779%	
	Ischemic colitis	18720	15.535%	25	38.462%	0.000
	diverticulitis	17530	5.654%	25	20.833%	0.000

We performed a multivariate analysis of the risk factors for CDI. After adjusting for all variables, multivariate analysis showed CDI was associated with ischemic colitis (OR = 2.06; 95% CI 1.59-2.65, p<0.001) (Table 4).

**Table 4:** Multivariate analysis of factors associated with C. difficile infection.

	Sig.	Odds Ratio	95% C.I.	
			Lower	Upper
Age	.000	.996	.994	.997
Race - White	.000	.629	.570	.694
Race - Black	.000	1.548	1.429	1.677
Race - Hispanic	.004	1.251	1.076	1.455
Medicare	.590	.974	.884	1.072
Medicaid	.000	1.253	1.160	1.353
Private insurance	.015	.854	.751	.970
Died	.000	.592	.489	.717
Length of Stay	.000	1.011	1.008	1.014
Anemia	.000	1.158	1.082	1.240
Congestive Heart Failure	.003	.843	.755	.942
Coagulopathy	.000	1.510	1.372	1.661
Hypertension	.000	1.160	1.086	1.239
Electrolyte imbalance	.000	9.963	9.370	10.593
Obesity	.483	1.034	.942	1.134
Renal failure	.063	1.089	.995	1.191
Weight loss	.000	1.510	1.375	1.657
Ischemic colitis	.000	2.055	1.590	2.656

## Discussion

Our study found that ischemic colitis was twice as likely to be seen in patients with CDI than in patients without C. difficile. In addition, C. difficile was 39% more likely to be seen in ischemic colitis than in acute diverticulitis patients. These findings support our hypothesis that tissue ischemia is a risk factor for CDI. As previously mentioned, very few previous studies have suggested a link between ischemia and CDI [4,5]. To our knowledge, there have been no studies analyzing the association between Ischemic colitis and CDI. This is the first of its kind.

The microbiology of C. difficile supports ischemia as a risk factor for this infection. C. difficile is an anaerobic gram-positive bacteria transmitted through the fecal-oral route via the ingestion of spores. Its spores are resistant to heat, acid, and antibiotics; in addition, its anaerobic nature allows C. difficile to thrive in low oxygen states [6]. Thus, in low oxygen and acidic conditions, C. difficile naturally survives while other bacteria die, leading to dysbiosis. Once in the colon, C. difficile converts from its spore form to its functional form, producing exotoxins (toxin A and toxin B) that act upon the intestinal epithelial cells and inflammatory cells causing tissue injury and diarrhea. The body normally protects itself from CDI through immune defense mechanisms in the epithelial layer of the intestine, producing antibodies to the toxins and toxic receptors, and by increasing IL-8. However, ischemia may damage these innate immune responses leading to increased infection risk.

The pathogenesis of ischemic colitis may also contribute to an increased risk of infectious colitis, including CDI. It involves decreased blood flow and ischemia of the mucosal layer, submucosal layer, and/or transmural involvement; this is followed by reperfusion injury. These states of ischemia and reperfusion injury lead to transient sub-epithelial hemorrhage, edema, ulceration of the mucosa, and occasionally permanent necrosis of the bowel wall. Thus, there are two mechanisms by which the risk of CDI may be increased in ischemic colitis. One is the presence of a low oxygen state, which allows the spores of C. difficile to survive while other normal flora perishes, causing dysbiosis. The other is the presence of edema and ulceration in the mucosa, which leads to a decrease in the intrinsic defense mechanism in the epithelium. This is similarly seen in ulcerative colitis, which is associated with an increased risk of CDI. Thus, the pathophysiology of C. difficile and ischemic colitis interprets the statistically significant association seen in our study.

Patients with ischemic colitis had 39% higher odds of contracting CDI than patients with acute diverticulitis. This is likely due to differences in pathogenesis, as ischemic colitis causes tissue ischemia, while diverticulitis tends to cause micro-perforations of the gut wall. To our knowledge, there has been no past data comparing the rates of CDI in both groups.

 Acute diverticulitis was chosen as a control group for several similarities that it shares with ischemic colitis while being considered a more benign disease. Acute diverticulitis is a major differential diagnosis of ischemic colitis. Demographics are similar in terms of age. Ischemic colitis patients are in their 60s-70s while the mean age at admission of acute diverticulitis is 63 years old [7]. In our study, patients with ischemic colitis had a mean age of 68-71 years. Both disorders affect the colon, where C. difficile primarily infects. Treatment is similar in both disease entities, consisting of supportive care (bowel rest and intravenous fluids) and broad-spectrum antibiotics.

Previous studies have shown that the use of antibiotics in uncomplicated diverticulitis may be equivocal to not using antibiotics, suggesting that the prevalence of infection in these patients is not significant [8]. There is also a lack of strong evidence to support the use of antibiotics in ischemic colitis [9]. That being said, empiric antibiotics are a commonly used therapy in both diverticulitis and ischemic colitis. They usually empirically cover gram-negative and anaerobes for 7-10 days [10,11]. Thus, one of the major risk factors for CDI, the use of antibiotics, is present in both ischemic colitis and diverticulitis patients. Empiric antibiotic regimens for both colonic diseases usually include PO or IV metronidazole, which would empirically cover for C. difficile infection. Thus, antibiotic use was not thought to disproportionately affect either group in this study. Unfortunately, we could not measure the proportion of patients that received antibiotics in either group.

Ischemic colitis patients are generally considered to have a worse prognosis compared to diverticulitis patients. The results of our study reflect this. In our study, mortality for ischemic colitis patients was 15%, while the mortality for diverticulitis patients was 1.4%. In comparison, the literature says mortality is 1%-3% [12] in uncomplicated diverticulitis. Meanwhile, mortality in ischemic colitis patients has been estimated to be approximately 22% [13], including 6% in medically managed patients and 39% for surgically managed patients [14]. Similarly, patients with ischemic colitis and CDI had higher mortality (7%) than patients with diverticulitis and CDI (0%). In addition, patients with ischemic colitis required a higher rate of surgeries (20%) than patients with diverticulitis (15%).

Our study also looked at other risk factors that may contribute to CDI. Older patients were more likely to have CDI; this is consistent with what has been found in the literature [15]. In addition, older patients are more likely to develop severe CDI [16]; we did not assess CDI severity in our study. Black and Hispanic patients were more likely than white patients to contract C. difficile. In our literature search, one study found that white patients had greater CDI rates than nonwhite patients, although these differences disappeared in a population for which healthcare access was presumed to be less racially biased [16]. In another study, CDI incidence was higher for white patients, but black race was independently associated with mortality and CDI [17]. Medicaid increased a patient's risk of developing CDI while having private insurance or Medicare did not correlate. No studies comparing CDI infection rates depending on insurance were found during the literature search. The cost of vancomycin and other antibiotics may contribute to this as patients may not be able to treat severe recurrent CDI [17].

Patients with chronic diseases such as anemia and hypertension had greater odds of having CDI in our study. No studies were found in our literature search associating these diseases to CDI; this may be a false positive given their high prevalence. Interestingly, renal failure was not associated with CDI and Congestive Heart failure decreased the odds of having CDI in our study. This is inconsistent with the literature. Studies have found that patients with CKD have a higher risk of CDI [18]. Heart failure has also been associated with higher rates of CDI [18]. In addition, the Charlson Comorbidity Index, which takes into account the presence of CHF and CKD among other diseases, was found to be a predictor of the need for hospitalization and complicated CDI [19]. Coagulopathy, electrolyte imbalance, and weight loss were also associated with CDI. This can be explained by sepsis and diarrhea.

It is important to note that CDI was associated with increased length of stay and increased risk of death in our study. Ischemic colitis patients with CDI had a mean length of stay of 22 days compared to 9 days in ischemic colitis patients without CDI. Diverticulitis patients with CDI had a mean length of stay of 8 days compared with a mean LOS of 5 days if they did not have CDI. This was expected as previous studies have suggested that CDI contributes to a longer length of stay [20]. Length of stay is very important because it proportionally increases healthcare resource expenditure. A retrospective study in the American Journal of Infection Control found that patients with CDI were associated with increased length of stay (by 4.7 days)-which led to increased attributable costs (by $7,286)-and increased mortality. These results are broadly similar to those of Kyne et al. [21] (attributable LOS, 3.6 days; attributable cost, $3,669) and Song et al. [20] (attributable LOS, 5.5 days; attributable cost, $6,326).

The main strength of our study is the large sample size which was obtained from a reliable representative database. There are few studies linking ischemic colitis to CDI, and no studies directly studying the two diseases were found during the literature search. Thus, this is the first study to do so. The use of multivariate analysis helped eliminate confounding variables. The observational characteristic of the study was beneficial as it is not an intervention and is of no harm to the patient.

Our study had a few limitations. For one, it is a retrospective study. Thus, it is not considered the highest level of evidence and is subject to confounders, such as the presence of comorbidities that may increase the risk of CDI. We performed a multivariable logistic regression analysis to assess the association between disease group and CDI while adjusting for confounders. Another limitation was that the diagnosis of the diseases and risk factors from the National inpatient survey is based on ICD-9 codes; thus, the correct data collection in this study is dependent on correct billing by physicians and staff. This could lead to record bias from under-reporting of diseases and variables. The possible underdiagnosis of ischemic colitis, as discussed previously, could also be a limitation. Given the retrospective nature and longitudinal nature of the study, it was not possible to establish whether the disease (CDI or diverticulitis) was causing an increase in the variables (such as LOS) or the variables caused an increase in the disease. We had no data on antibiotic regimens used or their duration, which is an important risk factor for CDI. Similarly, we had no data on the severity of CDI.

## Conclusions

Our study is novel in that it aimed to assess whether there is an association between tissue ischemia and CDI. The results of our study supported our hypothesis as we found that ischemic colitis was twice as likely to be seen in patients with CDI. The pathogenesis of C. difficile and ischemic colitis likely both contribute to this finding. CDI was associated with increased hospital stay and mortality. This undoubtedly leads to higher healthcare costs and utilization. The hope is that these findings will reintroduce the discussion and lead to more research on ischemic colitis as a risk factor for CDI. Increased awareness of ischemic colitis as a CDI risk factor can lead to improved surveillance strategies, more prompt treatment, more judicious use of antibiotics, and hopefully decrease healthcare utilization and costs in this patient population. This is important given the currently increasing economic burden of CDI in the healthcare system in the US and worldwide.
